# A Circadian Clock in the Olfactory Bulb Anticipates Feeding during Food Anticipatory Activity

**DOI:** 10.1371/journal.pone.0047779

**Published:** 2012-10-19

**Authors:** Nahum Nolasco, Claudia Juárez, Elvira Morgado, Enrique Meza, Mario Caba

**Affiliations:** 1 Doctorado en Ciencias Biomédicas, CIB, Universidad Veracruzana, Xalapa, Veracruz, México; 2 Centro de Investigaciones Biomédicas, Universidad Veracruzana, Xalapa, Veracruz, México; Yale School of Medicine, United States of America

## Abstract

Rabbit pups ingest food, in this case milk, once a day with circadian periodicity and are a natural model of food anticipatory activity. During nursing, several sensory systems receive information about properties of the food, one of them being the olfactory system, which has received little attention in relation to synchronization by food. In addition, the olfactory bulb has a circadian pacemaker that exhibits rhythms independently of the suprachiasmatic nucleus, but the biological functions of these rhythms are largely unknown. In the present contribution, we hypothesized that circadian suckling of milk synchronizes rhythms in the olfactory bulb. To this aim we explored by immunohistochemistry, rhythms of FOS and PER1 proteins, as indicators of activation and reporter of oscillations, respectively, through a complete 24-h cycle in periglomerular, mitral and granular cell layers of both the main and the accessory olfactory bulb. Subjects were 7-day-old rabbit pups scheduled to nurse during the night (02∶00 h) or day (10∶00 h), and also fasted subjects, to explore the possible persistence of oscillations. In the three layers of the main olfactory bulb, FOS was high at time of nursing, then further increased 1.5 h afterward, and then decreased to increase again in advance of the next nursing bout. This pattern persisted, without the postprandial increase, in fasted subjects with a shift in subjects nursed at 02∶00. PER1 was increased 2–8 h after nursing and this increase persisted in most cell layers, with a shift, in fasted subjects. In the accessory olfactory bulb we only observed a consistent pattern of FOS expression in the mitral cell layer of nursed subjects, similar to that of the main olfactory bulb. We conclude that the main olfactory bulb is synchronized during milk ingestion, but during fasting its oscillations perhaps are modulated by the suprachiasmatic nucleus, as proposed for rodents.

## Introduction

**Under daily food restriction many species** develop food anticipatory activity (FAA), which is considered a behavioral output of a putative food entrainable oscillator (FEO) [1]. Rather than being found in a single locus, it had been proposed that the FEO is a distributed system of interacting structures [2], [3]. During food ingestion several sensory systems receive information about properties of the food; one of them is the olfactory system. However, despite the fact that this structure plays an important role during meal ingestion [4], it has received little attention for its possible role in entrainment to cycles of food availability [**5**], [**6**]. One excellent model to explore the role of olfactory cues in synchronization of rhythms by food is the rabbit pup. These subjects show FAA, similar to adult rats under a schedule of food restriction [1]. On this basis, rabbit pups have been viewed as a natural model of FAA, as pups ingest food, in this case milk, just one time per day with a circadian periodicity [7], [8], [9]. In this process, the integrity of the olfactory system is crucial for survival as pups made anosmic by bilateral olfactory bulbs lesions are unable to suck milk and die of starvation [10].

Studies in the rat have demonstrated that the olfactory bulb (OB) contains an independent circadian pacemaker, as lesions of the suprachiasmatic nucleus (SCN) abolish circadian rhythms in locomotor behavior but not those of Period 1 (*Per1*) gene expression in the OB [11]. Moreover, there is a circadian rhythm in sensitivity to olfactory discrimination in the OB of the rat [12], [13] and the rabbit [14]. In the rabbit, mother’s milk contains a mammary pheromone (MP) that leads to localization of the nipples and ingestion of milk, and releases behavioral responses in the pup [15]. This behavior, as assessed by the frequency of searching and grasping responses, changes throughout the day, reaching a peak before the daily suckling bout [16]. The fact that this behavior is highest during FAA is consistent with its crucial role for pup’s survival, but also suggests physiological changes in the OB. The OB of rabbit pups expresses a diurnal rhythm of *Per1* and other clock genes [17], but is not known whether these rhythms can be entrained by external stimuli.

In the present experiment we hypothesized that circadian suckling of milk synchronizes the oscillator of the OB. To this aim we explored the effect of daily milk intake on the main (MOB) and accessory (AOB) olfactory bulbs by analyzing expression of FOS protein, as an index of neural activation [18], and protein PER1, product of the *per1* clock gene, as a reporter of circadian oscillations [19]. The expression of FOS and PER1 proteins were monitored during a complete 24 h cycle in pups nursed at two different times of day, one during the day and one during the night [20]. Furthermore, in order to explore possible persistence of oscillations, we additionally analyzed both proteins in another entire 24-h cycle in fasted subjects. The results confirmed that the OB has a circadian rhythm, in this case of the PER1 protein, and also of the FOS protein. In addition, we report that the rhythms of these proteins are synchronized by milk ingestion, as this effect shifts in parallel to timing of suckling. **Finally, we found that the MOB shows a more consistent pattern than the AOB, as we observed an entraining of the three cell layers in the MOB but not in the AOB.**

## Materials and Methods

### Animals and General Housing Conditions

All experimental procedures were approved by the Ethics Committee of Universidad Veracruzana, in accordance with the procedures of the National Guide for the Production, Care and Use of Laboratory Animals (Norma Oficial Mexicana NOM-062-ZOO-1999), which complies with international guidelines of the Society for Neuroscience on the ethical use of animals. All efforts were made to minimize suffering. New Zealand white female rabbits bred in our colony in Xalapa, México, were housed under controlled light conditions (12/12 h light/dark cycle, lights on at 07∶00 h), stable temperature conditions (23±2°C) and provided with rabbit chow (Purina) and water *ad libitum*. The pregnant females were kept individually in steel cages and monitored daily from day 28 of pregnancy until delivery. The cage consisted of three compartments, one for the mother, one for the nest and a tunnel between them; this latter had a sliding door in order to control the access of the mother to the nest [18]. In the last week of pregnancy, each doe was provided with about 100 g of straw for building of the nest; then, just before parturition, she covered the nest with fur from her belly. On the day of parturition (PD0), litters were adjusted to 6–8 pups and remained in the maternal nest, in constant darkness and undisturbed for the entire experiment. To determine that pups were synchronized to daily food intake, their locomotor behavior was monitored in the nest with a detector sensitive to pup’s movements through the infrared radiations emitted by their body [9], [21].

### Experimental Groups and Sampling

On PD0 the door between the mother’s compartment and tunnel was locked, and starting the next day (PD1) was opened either at 02∶00 h or 10∶00 h from PD1 to PD7. At PD7 pups were assigned to two groups, Restricted Food (RF) 02∶00 or RF10∶00, on the basis of their time of nursing. Nursing time was considered zeitgeber time (ZT) 0, and pups were randomly killed at ZT0, at ZT1.5 and then at 4-h intervals after nursing, at ZT04, ZT08, ZT12, ZT16 and ZT20 (n = 4 at each time point). To explore persistence of possible oscillations additional subjects were fasted (restricted feeding-fasted; RF-F) at PD8, and were killed starting after expected nursing and then at similar intervals as nursed subjects (n = 4 at each time point), either in pups nursed at 02∶00 h (RF-F02∶00) or 10∶00 h (RF-F10∶00). Rabbit pups were euthanized with an overdose of sodium pentobarbital (20 mg per pup, intraperitoneal) and were perfused transcardially with saline solution (0.9%), followed by 4% paraformaldehyde in phosphate buffer (PB, pH 7.4). We used the olfactory bulbs from rabbits whose brainstem tissue was reported previously [20]. The olfactory bulbs were removed immediately after perfusion, cryoprotected successively in 10, 20, and 30% sucrose in phosphate buffer 0.1 M (PB) and then sectioned coronally at 50 µm with a cryostat (Microm).

### Immunohistochemistry

Serial sections were collected in PB 0.1 M; every two of four sections was used for labeling of FOS or PER1 as described below, following protocols previously established in rabbit brain [20], [21], [22]. Tissue was washed in PB four times, 5 min each to remove excess of aldehydes and then exposed for 10 min in 0.5% hydrogen peroxide solution to eliminate endogenous peroxidase activity. Free floating sections were incubated in PER1 antibody raised in goat (sc-7724, Santa Cruz Biotechnology, Santa Cruz, CA, USA) or in c-Fos antibody raised in goat (sc-52G, Santa Cruz Biotechnology, Santa Cruz, CA, USA) both diluted at 1∶2000, in 3% normal horse serum and 0.3% Triton X-100 (Sigma, St. Louis MO, USA), for 48 h at 4°C. It was followed by incubation in secondary antibody, biotinylated horse anti-goat diluted at 1∶200 (Vector Laboratories, Burlingame, CA, USA) in PB and 0.3% Triton X-100 for 1 h at room temperature. Then, sections were incubated in avidin-biotin-horseradish peroxidase complex diluted at 1∶200 (Vector Laboratories) in PB and 0.3% Triton X-100 for 1 h at room temperature. Between incubations, tissue was washed four times for 10 min each in PB. Both FOS and PER1 antibody-peroxidase complex was visualized with a solution of 0.05% diaminobenzidine (DAB; Polysciences, Warrington, PA, USA) in the presence of nickel sulfate (10 mg/ml, Fisher Scientific, NJ, USA), cobalt chloride (10 mg/ml, Fisher Scientific) and 0.01% hydrogen peroxide to obtain a black-purple precipitate. After 10 min, tissue was transferred to PB to stop the reaction. Sections were mounted onto gelatin-subbed slides, dehydrated and coverslipped with Permount. In all cases, tissue sections from subjects of each of the different time points were processed together. Control sections were processed as above, but with the primary antibody omitted. Additionally, sc-7724 PER1 and sc-52 FOS antibodies have been previously validated and characterized in rabbit tissue [20].

### Cell Count

In order to quantify PER1- and FOS- immunoreactive (IR) positive cells in the MOB, one representative section containing the medial level of the AOB was selected **at plate 17 of a rabbit brain atlas** [23]. We analyzed PER1- and FOS-IR in the periglomerular, mitral and granular cell layers [23], [24]. In the MOB we selected an area of 0.022 mm^2^, 0.0134 mm^2^ and 0.0487 mm^2^ for the periglomerular, mitral and granular cell layers, respectively. In the AOB we analyzed an area of 0.0130 mm^2^ for the periglomerular layer and 0.0217 mm^2^ for the mitral and granular cell layers. Then we used an image analysis system to measure optic density in a surface area (Image Pro Plus v. 5; Media Cybernetics, Silver Spring, MD, USA), quantifying the number of cell nuclei in the visual field delimited by the drawn contour surrounding each layer **according to an automated cell count** [**25**] **. This method detects objects (cells) on basis of a group of pixels with values of optical density above the threshold, which are expressed as number of cells.** In each section, the detection level (grey scaling) was chosen above the background level. Cells positive to PER1- and FOS-IR were counted unilaterally in the left MOB and AOB by two observers blind to experimental conditions. Selected images were digitized at 10X magnification using a computerized image system (Image Pro Plus v. 5) attached to a light microscope (Olympus BX41).

### Statistical Analysis

A two-way ANOVA for the main effects of group condition, time factor and the interaction between group and time was carried out, in which the dependent variables were the state of feeding, either nursing or fasting, while the independent variable was the time at which pups were nursed or fasted. This analysis was followed by a post hoc Tukey for 2×2 comparison of equivalent time in both conditions. Statistical analyses were performed using Sigma Stat Statistical Software version 3.5. Probability levels of *P*<0.05 were considered significant. Values given are means ± SEM.

## Results

### FOS-IR in Periglomerular, Mitral and Granular Cell Layers in the MOB

**Figure 1:**
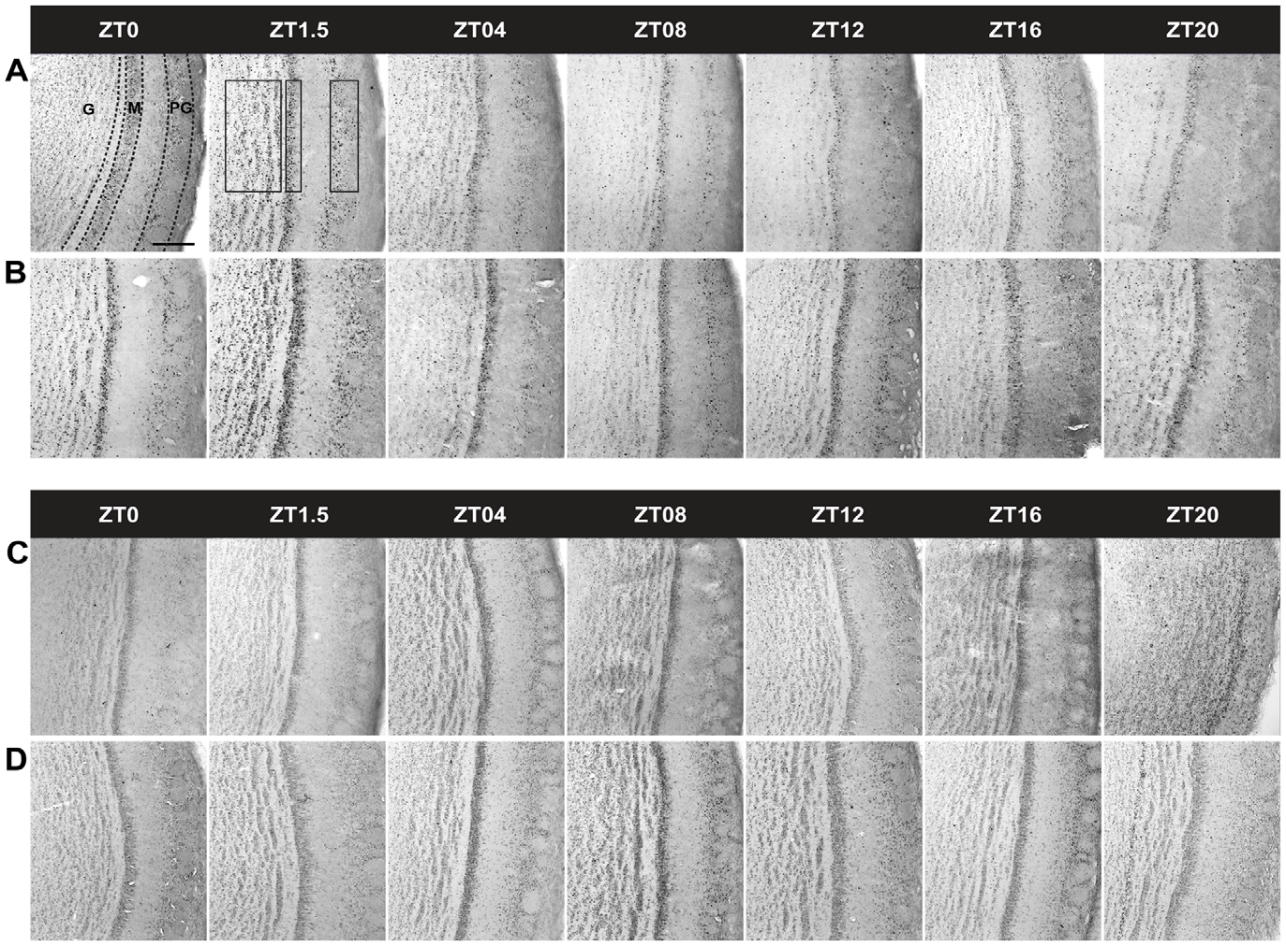
FOS and PER1 expression in the main olfactory bulb of 7–8-day-old rabbit pups. Photomicrographs showing expression of FOS (A, B) and PER1 (C,D) in nursed subjects at 02∶00 (A,C) or 10∶00 (B,D) h. Note that FOS is high at time of nursing (ZT0) and 1.5 h later (ZT1.5), and PER1 is high 8 h after nursing (ZT08). Dotted lines delimit the layers. G, Granular cell layer; M, Mitral cell layer; PG, periglomerular cell layer. **Rectangles** represent the counted area. Scale bar, 100 μm.

**Figure 2:**
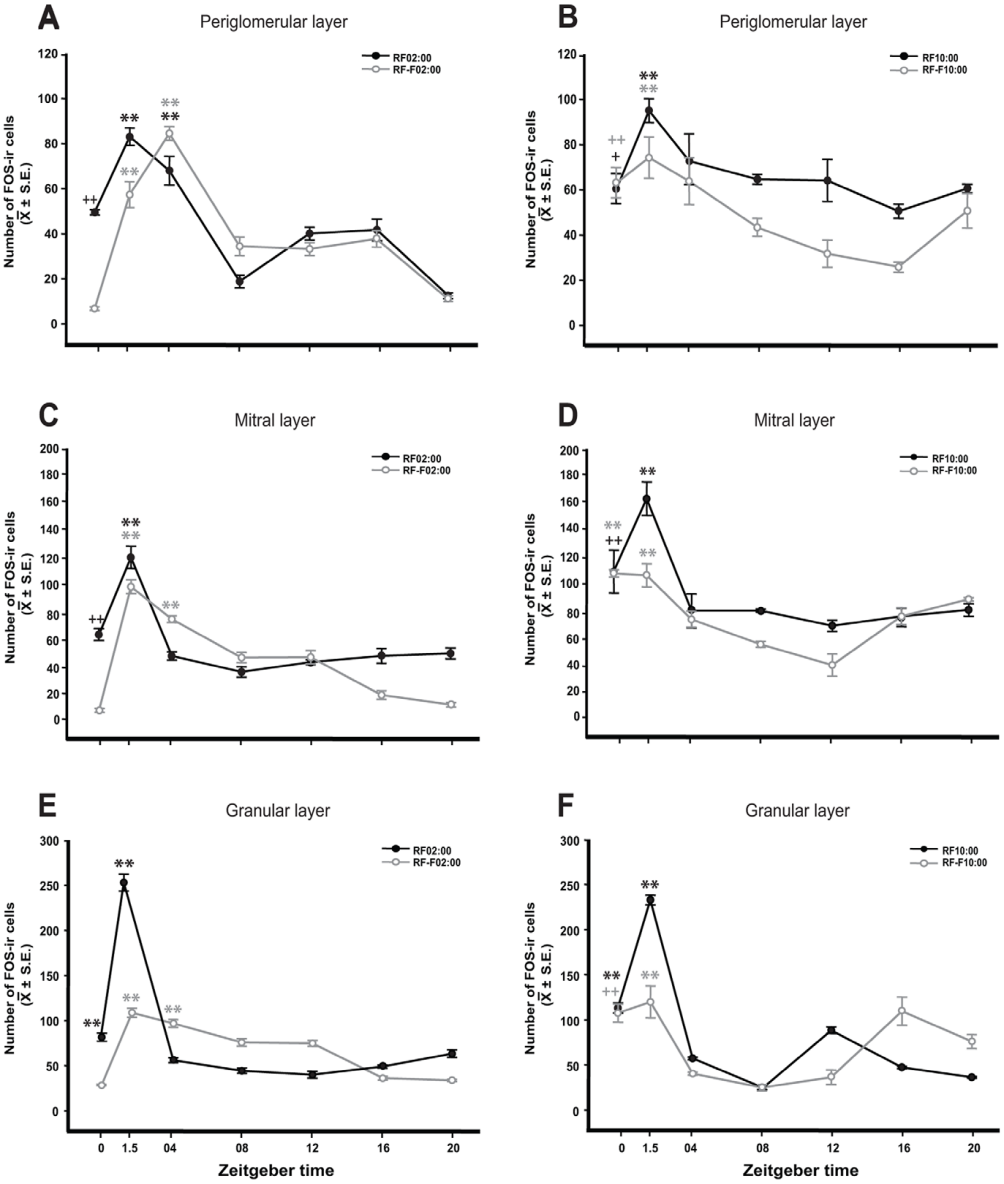
FOS expression in the main olfactory bulb of 7–8-day-old rabbit pups nursed (RF02∶00; RF10∶00) and fasted (RF-F02∶00; RF-F10∶00). Graphs show FOS expression in the periglomerular, mitral and granular cell layers in pups nursed and fasted during the night (A, C, E) or during the day (B, D, F). Note that all layers exhibit an increase of FOS expression at the time of nursing and 1.5 h later. X axis indicates Zeitgeber time (ZT0 is time of nursing) in RF animals, or hours after scheduled nursing in un-nursed animals in RF-F animals. Values are mean ± SEM. * Significant difference between highest and lowest values in each group. ^+^ Significant difference of ZT0 time point or same time point in un-nursed animals against the lowest values. +, P<0.05. **, ++, P<0.01(see text for details).

In Fig. 1 we present FOS and PER1 expression in the MOB **of nursed** 7–8-day-old rabbit pups. In both night and day nursed subjects FOS expression followed a rhythm in the three cell layers, which shifted in parallel to timing of nursing (Fig. 2). Values were high at time of nursing, then 1.5 h later increased further, and then decreases to lower values. In fasted RF10∶00 subjects we observed a similar pattern, but in RF02∶00 subjects the increase at the expected time of nursing was delayed for 1.5 h.

### FOS-IR in Periglomerular Cell Layer

#### Subjects nursed at 02∶00 h

Two-way ANOVA indicated that in nursed and fasted pups FOS expression in the periglomerular layer in MOB varied significantly with group condition (F_1,55_ = 12.98, *P*<0.001), time factor (F_6,55_ = 86.15, *P*<0.001), and the interaction between feeding condition and time (F_6,55_ = 17.68, *P*<0.001; Fig. 2A). In the RF02∶00 group, the highest FOS expression at ZT1.5 and ZT04 was significantly different to values at ZT0, ZT08, ZT12, ZT16 and ZT20 (*P*<0.001 in all cases). Additionally, ZT0 value was higher than ZT08 and ZT20 values (*P*<0.001 in both cases). In the RF-F02∶00 group, the highest expression of FOS was observed at ZT1.5 and ZT04 (*P*<0.001 against all values).

#### Subjects nursed at 10∶00 h

Quantitative analysis indicated that in nursed and fasted pups FOS expression in the periglomerular layer in MOB varied significantly with group condition (F_1,55_ = 19.73, *P*<0.001), time factor (F_6,55_ = 9.33, *P*<0.001), but not in the interaction between feeding condition and time (F_6,55_ = 1.46, *P* = 0.215; Fig. 2B). In the RF10∶00 group, the highest value at ZT1.5 was significantly different than values at ZT16 (*P*<0.001), ZT0, ZT20 (*P* = 0.01 in both cases), ZT08 and ZT12 (*P* = 0.04 in both cases). In addition ZT0 was higher than value at ZT16 (P = 0.05). In RF-F10∶00, the highest expression of FOS at ZT1.5 was significantly different to values at ZT08 (*P* = 0.043), ZT12 (*P* = 0.002) and ZT16 (*P*<0.001). Additionally, ZT0 and ZT04 values were higher than values at ZT12 (*P* = 0.03 in both cases) and ZT16 (*P* = 0.007 in both cases).

### FOS-IR in Mitral Cell Layer

#### Subjects nursed at 02∶00 h

Two-way ANOVA indicated that in nursed and fasted pups FOS expression in the mitral layer in MOB varied significantly with group condition (F_1,55_ = 43.166, *P*<0.001), time factor (F_6,55_ = 88.45, *P*<0.001), and the interaction between feeding condition and time (F_6,55_ = 24.93, *P*<0.001; Fig. 2C). In the RF02∶00 group, the highest FOS expression at ZT1.5 was significantly different to remaining time point values (*P*<0.001 in all cases). Additionally, the FOS expression at ZT0 was higher than values at ZT08 (*P*<0.001) and ZT12 (*P = *0.02). In the RF-F02∶00 group the highest expression of FOS was observed at ZT1.5 and ZT04 (*P*<0.001 against all values).

#### Subjects nursed at 10∶00 h

Quantitative analysis indicated that in nursed and fasted pups FOS expression in the mitral layer in MOB varied significantly with group condition (F_1,55_ = 4.65, *P* = 0.037), time factor (F_6,55_ = 17.88, *P*<0.001), and the interaction between feeding condition and time (F_6,55_ = 2.567, *P* = 0.033; Fig. 2D). In the RF10∶00 group, the highest value at ZT1.5 was significantly different than values at ZT04, ZT12, ZT16 (P<0.001 in all cases), ZT08 and ZT20 (*P* = 0.006 in both cases). Additionally, ZT0 value was higher than value at ZT12 (*P*<0.01). In RF-F10∶00, the highest expression of FOS at ZT0 and ZT1.5 was significantly different to values at ZT08, ZT12 (*P*<0.001 in both cases), ZT04 and ZT16 (*P*<0.05 in both cases). Additionally, ZT20 value was higher than value at ZT08 and ZT12 (*P*<0.001 in both cases).

### FOS-IR in Granular Cell Layer

#### Subjects nursed at 02∶00 h

Two-way ANOVA indicated that in nursed and fasted pups FOS expression in the granular layer in MOB varied significantly with group condition (F_1,55_ = 3.42, *P* = 0.071), time factor (F_6,55_ = 85.98, *P*<0.001), and the interaction between feeding condition and time (F_6,55_ = 79.20, *P*<0.001; Fig. 2E). In the RF02∶00 group, the highest FOS expression at ZT0 and ZT1.5 was significantly different to remaining time point values (*P*<0.001 in all cases, except ZT0 vs ZT20 *P* = 0.011). Additionally, ZT20 value was higher than values at ZT08, ZT12 (*P*<0.001 in both cases), and ZT16 (*P* = 0.016). In the RF-F02∶00 group, the highest expression of FOS was observed at ZT1.5 and ZT04 (*P*<0.001 against all values, except ZT04 vs ZT08 *P* = 0.011).

#### Subjects nursed at 10∶00 h

Quantitative analysis indicated that in nursed and fasted pups FOS expression in the granular layer in MOB varied significantly with group condition (F_1,55_ = 8.7, *P* = 0.005), time factor (F_6,55_ = 80.2, *P*<0.001), and the interaction between feeding condition and time (F_6,55_ = 26.95, *P*<0.001; Fig. 2F). In the RF10∶00 group, the highest value at ZT1.5 was significantly different to remaining time point values (*P*<0.001 in all cases). Additionally, ZT0 value was higher than values at ZT04, ZT08, ZT16, and ZT20 (*P*<0.001 in all cases). In RF-F10∶00, the highest expression of FOS at ZT1.5 was significantly different to values at ZT04, ZT08, ZT12, and ZT20 (*P*<0.001 in all cases). Additionally, ZT0, ZT16, and ZT20 were higher than values at ZT04, ZT08, and ZT12 (*P*<0.001 in all cases, except ZT20 vs ZT12 *P* = 0.016 and ZT20 vs ZT04 *P* = 0.04).

### PER1-IR in Periglomerular, Mitral and Granular Cell Layers in the MOB

**Figure 3:**
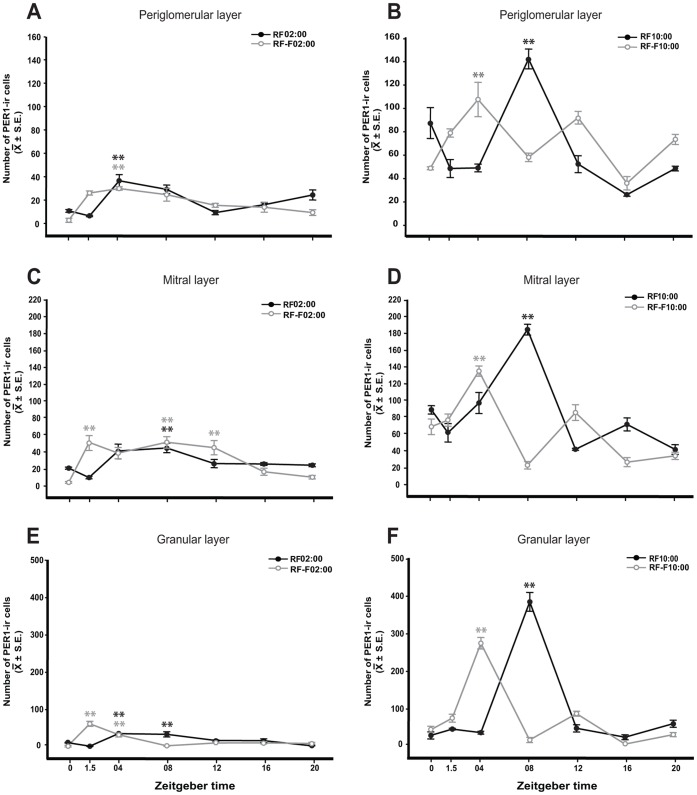
PER1 expression in the main olfactory bulb of 7–8-day-old rabbit pups nursed (RF02∶00; RF10∶00) and fasted (RF-F02∶00; RF-F10∶00). Graphs show PER1 expression in the periglomerular, mitral and granular layers in pups nursed and fasted during the night (A, C, E) or during the day (B, D, F). The acrophase of PER1 expression was 4–8 h after nursing time. Values are mean ± SEM. * Significant difference between highest and lowest values in each group. **, P<0.01 (see text for details).

In both night and day nursed subjects, PER1 expression increased 4–8 h after nursing in the three cell layers, shifting in parallel to timing of nursing (Fig. 3). However, notably, the increase was of higher magnitude in night versus day-nursed pups. In fasted subjects we also observed an increase but was advanced 4–5.5 h with respect to that in nursed subjects; this was more evident in day-nursed pups.

### PER1-IR in Periglomerular Cell Layer

#### Subjects nursed at 02∶00 h

Two-way ANOVA indicated that in nursed and fasted pups PER1 expression in the periglomerular layer in MOB not varied significantly with group condition (F_1,55_ = 0.838, *P* = 0.365), but with the time factor (F_6,55_ = 16.922, *P*<0.001) and the interaction between feeding condition and time (F_6,55_ = 6.748, *P*<0.001; Fig. 3A) varied significantly. In the RF02∶00 group, the highest PER1 expression at ZT04 was significantly different to values at ZT0, ZT1.5, ZT12 and ZT16 (*P*<0.001 in all cases). In the RF-F02∶00 group, the highest expression of PER1 was observed at ZT04, significantly different to values at ZT0, ZT20 (*P*<0.001 in both cases), ZT16 (*P* = 0.009) and ZT12 (*P* = 0.03).

#### Subjects nursed at 10∶00 h

Quantitative analysis indicated that in nursed and fasted pups PER1 expression in the periglomerular layer in MOB not varied significantly with group condition (F_1,55_ = 2.06, *P* = 0.159), but the time factor (F_6,55_ = 16.68, *P*<0.001) and the interaction between feeding condition and time (F_6,55_ = 25, *P*<0.001; Fig. 3B) varied significantly. In the RF10∶00 group, the highest value at ZT08 was significantly different to remaining time point values (*P*<0.001). In RF-F10∶00, the highest expression of PER1at ZT04 was significantly different to values at ZT0, ZT08, ZT16 (P<0.001) and ZT20 (P = 0.022).

### PER1-IR in Mitral Cell Layer

#### Subjects nursed at 02∶00 h

Two-way ANOVA indicated that in nursed and fasted pups PER1 expression in the mitral layer in MOB not varied significantly with group condition (F_1,55_ = 1.26, *P* = 0.267), but the time factor (F_6,55_ = 11.69, *P*<0.001), and the interaction between feeding condition and time (F_6,55_ = 6.93, *P*<0.001; Fig. 3C) varied significantly. In the RF02∶00 group, the highest PER1expression at ZT08 was significantly different than values at ZT0 (*P* = 0.032) and ZT1.5 (P<0.001). In the RF-F02∶00 group, the highest expression of PER1 was observed at ZT1.5, ZT08 and ZT12 (*P*<0.001 against all values, except ZT12 vs ZT16 *P* = 0.018).

#### Subjects nursed at 10∶00 h

Quantitative analysis indicated that in nursed and fasted pups PER1 expression in the mitral layer in MOB varied significantly with group condition (F_1,55_ = 24.49, *P*<0.001), time factor (F_6,55_ = 28.9, *P*<0.001), and the interaction between feeding condition and time (F_6,55_ = 44.56, *P*<0.001; Fig. 3D). In the RF10∶00 group, the highest value at ZT08 was significantly different to remaining time point values (*P*<0.001in all cases). In RF-F10∶00, the highest expression of PER1 at ZT04 was significantly different to remaining time point values (*P*<0.001in all cases).

### PER1-IR in Granular Cell Layer in MOB

#### Subjects nursed at 02∶00 h

Two-way ANOVA indicated that in nursed and fasted pups PER1 expression in the granular layer in MOB not varied significantly with group condition (F_1,55_ = 0.52, *P* = 4.72), but the time factor (F_6,55_ = 15.24, *P*<0.001), and the interaction between feeding condition and time (F_6,55_ = 30.13, *P*<0.001; Fig. 3E) varied significantly. In the RF02∶00 group, the highest PER1 expression at ZT04 and ZT08 was significantly different to remaining time point values (*P*<0.001). In the RF-F02∶00 group, the highest expression of PER1 at ZT1.5 was significantly different to remaining time point values (*P*<0.001 in all cases). Additionally, the PER1 expression at ZT04 was higher than values at ZT0, ZT08 (*P*<0.001 in both cases), ZT12 (*P* = 0.016), ZT16 and ZT20 (*P* = 0.004).

#### Subjects nursed at 10∶00 h

Quantitative analysis indicated that in nursed and fasted pups PER1 expression in the granular layer in MOB not varied significantly with group condition (F_1,55_ = 0.21, *P* = 0.644), but the time factor (F_6,55_ = 14.72, *P*<0.001) and the interaction between feeding condition and time (F_6,55_ = 22.08, *P*<0.001; Fig. 3F) varied significantly. In the RF10∶00 group, the highest value at ZT08 was significantly different to remaining time point values (*P*<0.001in all cases). In RF-F10∶00, the highest expression of PER1 at ZT04 was significantly different to remaining time point values (*P*<0.001).

### FOS- and PER1-IR in Periglomerular, Mitral and Granular Cell Layers in the AOB

**Figure 4:**
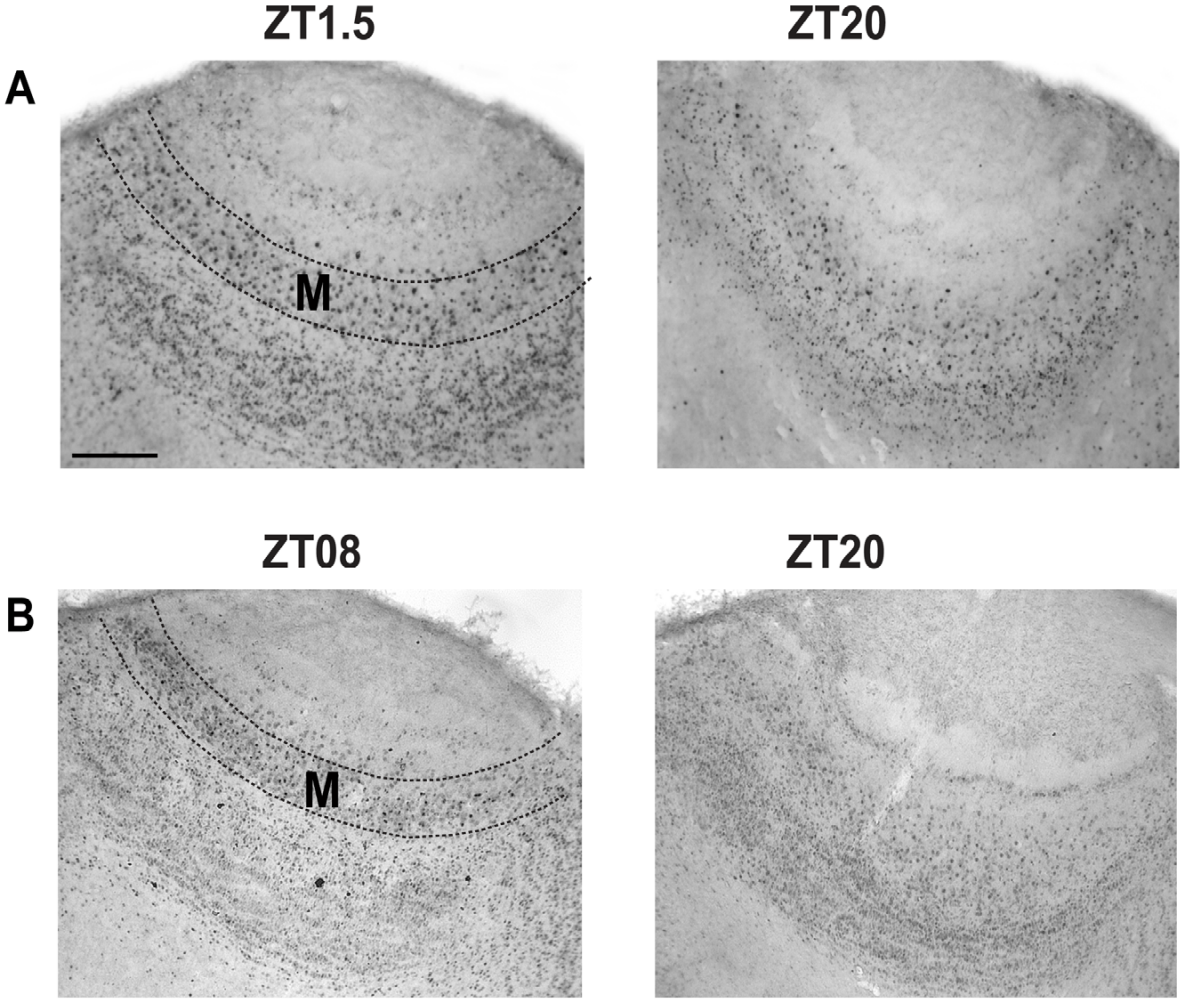
FOS and PER1 expression in the accessory olfactory bulb of 7–8-day-old rabbit pups nursed at 10∶00 h. Photomicrographs showing expression of FOS (A) and PER1 (B) at times of high (ZT1.5; ZT08) and low (ZT20) proteins expression. Dotted lines delimit the mitral cell layer (M). Scale bar, 100 μm.

**Figure 5:**
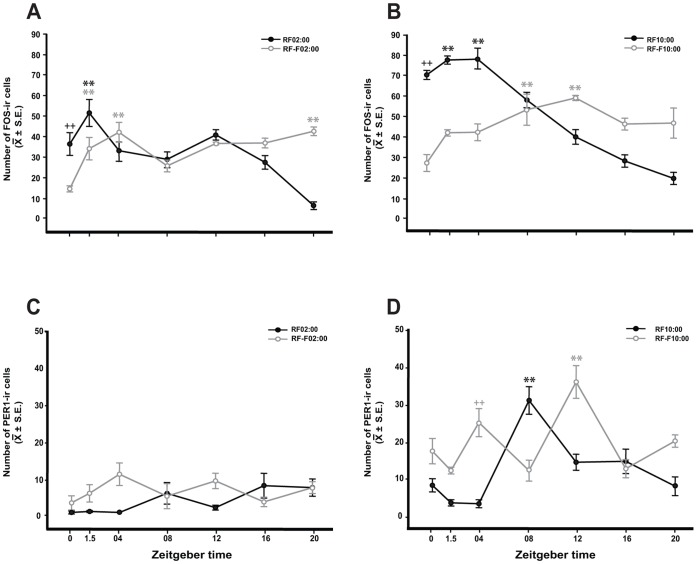
FOS and PER1 expression in the mitral layer of the accessory olfactory bulb of 7–8-day-old rabbit pups nursed (RF02∶00; RF10∶00) and fasted (Rf-F02∶00; RF-F10∶00). Graphs shows FOS (A, B) and PER1 (C, D) in the mitral cell layer in pups nursed and fasted during the night (A, C) or during the day (B, D). Note that FOS expression is high at the time of nursing and increases further 1.5 h later. PER1 expression has a rhythm only in RF10∶00 group with an increase 8 h after nursing. Values are mean ± SEM. * Significant difference between highest and lowest values in each group. ^+ +^ Significant difference of ZT0 and ZT04 time points against the lowest values. **, ++, P<0.01 (see text for details).

In the AOB, in both night and day nursed and fasted subjects FOS and PER1 proteins were expressed without an apparent rhythm in the periglomerular and granular cell layers. Timing of nursing did not seem to affect the induction of these proteins as revealed by their different levels at the same time point in night versus day nursed subjects (**Fig. S1**). On the contrary, in the mitral cell layer we observed an effect of nursing on both FOS and PER1 (Fig. 4), only in nursed subjects, which shows an increase at the time and after nursing. In relation to PER1, we only observed an induction of this protein in day-, but not in night-nursed subjects (Fig. 5).

### FOS-IR in Mitral Cell Layer in AOB

#### Subjects nursed at 02∶00 h

Two-way ANOVA indicated that in nursed and fasted pups FOS expression in the mitral layer in AOB not varied significantly with group condition (F_1,55_ = 0.36, *P*<0.548), however, we observed an effect in time factor (F_6,55_ = 7.07, *P*<0.001), and the interaction between feeding condition and time (F_6,55_ = 12.97, *P*<0.001; Fig. 4A). In the RF02∶00 group, the highest FOS expression at ZT1.5 was significantly different values at ZT16, ZT20 (*P*<0.001 in both cases), ZT04 (*P* = 0.002), and ZT08 (*P* = 0.019). Additionally, value at ZT0 was different from the lowest value at ZT20 (*P*<0.001). In the RF-F02∶00 group, the highest expression of FOS was observed at ZT20, different to values at ZT0 (*P*<0.001) and ZT08 (*P* = 0.048). Additionally, value at ZT04 (*P*<0.001) was different from the lowest value at ZT0.

#### Subjects nursed at 10∶00 h

Quantitative analysis indicated that in nursed and fasted pups FOS expression in the mitral layer in AOB varied significantly with group condition (F_1,55_ = 12.87, *P*<0.001), time factor (F_6,55_ = 13.22, *P*<0.001), and the interaction between feeding condition and time (F_6,55_ = 26.58, *P*<0.001; Fig. 4B). In the RF10∶00 group, the highest values at ZT1.5 and ZT04 were significantly different than values at ZT12, ZT16, ZT20 (P<0.001 in all cases), and ZT08 (*P* = 0.02 in both cases). Additionally, value at ZT0 was higher than values at ZT12, ZT16, and ZT20 (*P*<0.001 in all cases). In RF-F10∶00, the highest expression of FOS at ZT08 and ZT12 was significantly different to value at ZT0 (*P*<0.001 in both cases).

### PER1-IR in Mitral Cell Layer in AOB

#### Subjects nursed at 02∶00 h

Two-way ANOVA indicated that in nursed and fasted pups PER1 expression in the mitral layer in AOB varied significantly with group condition (F_1,55_ = 6.15, *P* = 0.017) and the interaction between feeding condition and time (F_6,55_ = 2.78, *P*<0.023), but the time factor (F_6,55_ = 1.4, *P*<0.238; Fig. 4C) not affected significantly. Any time point was different in paired comparison.

#### Subjects nursed at 10∶00 h

Quantitative analysis indicated that in nursed and fasted pups PER1 expression in the mitral layer in AOB varied significantly with group condition (F_1,55_ = 26.816, *P*<0.001), time factor (F_6,55_ = 9.25, *P*<0.001), and the interaction between feeding condition and time (F_6,55_ = 13.73, *P*<0.001; Fig. 4D). In the RF10∶00 group, the highest value at ZT08 was significantly different to remaining time point values (*P*<0.001in all cases). In RF-F10∶00, the highest expression of PER1 at ZT12 was significantly different to values at ZT0, ZT1.5, ZT08 (*P*<0.001in all cases), and ZT20 (*P*<0.003). Additionally, value at ZT04 was higher than values at ZT1.5, ZT08 (*P* = 0.026), and ZT16 (*P* = 0.036).

## Discussion

Previous studies have indicated that the OB expresses circadian rhythms and that this structure contains an independent circadian pacemaker, which is entrained to the light/dark cycle. In the present study we demonstrate, for the first time, that circadian rhythms in the OB are entrained to mealtime, which suggests a biological function for this pacemaker in FAA, especially as it is seen in subjects that are unable to receive light cues at this age.

In the adult rat, explant cultures of the OB show circadian rhythms in firing rate and *Period1-luciferase*, which are intrinsic to this tissue, as they do not depend on the SCN [11], [26]. Based on this, an important question was to know whether these rhythms could have a functional consequence. Indeed, the response of the OB, assessed by the expression of c-Fos protein, to cedar oil in intact rats is rhythmic. Approximately four times as many cells were labeled during subjective night compared with the subjective day when the animals were exposed to that olfactory stimulus. Moreover, these oscillations persist in constant darkness in SCN lesioned subjects [27]. These results are in agreement with previous experiments, which show that in the rat, there is also a circadian rhythm in response to a neutral odor, cedar wood oil, and to a biological relevant odor, the odor of the red fox. In both cases the response, also assessed by c-Fos expression, was higher in the subjective night than in the subjective day [12], [28]. Together this evidence reveals a circadian modulation of the sensory sensitivity of the OB. In agreement, the present results also reveal circadian rhythms in OB, but in addition show that these rhythms can be entrained by an external stimulus, specifically food ingested during suckling.

What is the precise entraining signal? One of the key components of rabbit mother’s milk is 2–methylbut-2-enal, known as MP [15]. As mentioned previously, MP detection by the OB is crucial for rabbit pups survival as anosmic newborn subjects die of starvation. Moreover, the behavioral response of rabbit pups to MP at PD5 changes in relation to prandial state. It is highest just before the daily suckling bout episode, then sharply decreases after suckling to return 6 h later to levels before nursing [16]. This evidence suggests that nursing time may entrain OB responsiveness to MP, including physiological mechanisms mediating that response at the cellular level. In fact, as shown in results by using a strategy of scheduling nursing at two different times we were able to demonstrate that timing of nursing indeed entrains both cellular FOS and PER1 rhythms in the OB.

However, timing of nursing differentially affects rhythms of FOS in the OB. In both RF02∶00 and RF10∶00 subjects, values follow a similar pattern through the cycle, shifting in parallel, showing an anticipatory increase at the time of nursing and then a further, postprandial, increase. However in fasted subjects, after skipping one nursing bout, FOS in RF-F10∶00 peaks at the time of the scheduled nursing, but in RF-F02∶00 FOS increases around 1.5–4 h after nursing expected. Restated, in both cases the rhythm is maintained but in RF-F02∶00 subjects this increase delays 1.5 h. We do not know the reason for this difference but perhaps is due to an effect of the SCN. As mentioned, in rodents there is a circadian response to cedar oil, but in SCN-lesioned mice the evoked response of c-Fos by this odor in the OB persisted but shifted to 12 h out of phase with that in intact subjects [27]. These authors suggest that there is an entraining signal of the SCN on OB, which is lost after ablation of this nucleus so the OB likely free-runs. Previously we reported that the rabbit pup’s SCN has a robust circadian rhythm in both FOS and PER1 proteins and moreover the locomotor behavior, a rhythm controlled by the SCN, is significantly higher before and after suckling in RF10∶00 in comparison to RF02∶00 subjects of the same age as present contribution [20]. On this basis, we propose that the shift of FOS rhythm in fasted subjects perhaps is due to a differential day- versus night-action of SCN on OB [12] and also similar as that reported in rodents in relation to the SCN with the dorsomedial nucleus of the hypothalamus [29], [30]. In support of this, indirect afferents from the SCN to OB had been described [31], [32]. Besides that, a light pulse evokes *Per1* and *Per2* mRNA expression in the OB, which indicates that receives photic information, perhaps through the SCN [33]. Future studies should address this possibility in the rabbit pup.

In relation to PER1, we also found a clear difference in RF10∶00 in comparison to RF02∶00 subjects. Previously we and others have reported that in several areas of the brain this protein is induced and reaches a peak 4–12 h after receiving a stimulus [19], [20], [21], [22], [34], [35]. *Per1* is a clock gene that endogenously oscillates in many brain regions, but its rhythm is phase shifted by restricted feeding. For this reason, assessment of rhythms of this gene and its protein is commonly used as an index of synchronization by food [19], [34], [35]. Similar to our previous publications, we found an induction of PER1 in the OB of nursed subjects, but it is noteworthy that RF10∶00 subjects display far more PER1 expression, as reflected by numbers of cells, than RF02∶00 subjects. Interestingly fasted subjects also show a peak of PER1 but this is advanced in most layers in both day and night-fasted subjects, in comparison to that of nursed subjects. We consider this is an interesting result. PER1 increase after nursing suggest that MOB is important for synchronization when food is present, as not all nuclei related to food intake, e.g. in the brainstem, are synchronized by scheduled meal [21]. But at the same time seems that lack of food also induces PER1 increase, perhaps indicating lack of food to other brain regions, similar to a previous report [21]. Although RF-F10∶00 group shows a secondary PER1 peak, both fasted groups shows this increase 1.5–04 h after the expected time of nursing. While we do not know the reason for this differences, perhaps is partially due to a day versus night influence from the SCN on OB which should be explored in future experiments.

In contrast to the three cell layers of the MOB, results in the AOB were less consistent and we only found a clear rhythmic pattern in the mitral cell layer, **but not in the periglomerular and granular cell layers (Supporting information, Fig. S1).** **In the mitral cell layer** there is also an induction of FOS, both in RF02∶00 and RF10∶00 subjects, which drops to lowest levels 20 h after nursing. But unlike the main olfactory bulb, in fasted subjects we did not see a clear pattern in both groups. Also in relation to PER1, only RF10∶00 subjects show a clear induction of this protein 8 h after suckling, which does not persist, in fasted subjects. In RF02∶00 pups this protein is also expressed but without a clear pattern. As already mentioned, in the periglomerular and granular cell layers PER1 was expressed but without an apparent rhythm (**Fig. S1**). Taken together, these results suggest that the accessory olfactory system does not play a role as important as that of the main olfactory system during suckling of milk. This assumption is not surprising as MP presented with a glass rod to 4 days old rabbit pups elicited widespread FOS protein in the MOB, while the AOB exhibits a negligible staining [24]. In addition, surgical removal of the vomeronasal organ in newborn rabbits does not affect the pup’s ability to respond to the pheromone released by a lactating mother and the ability to suck milk during nursing is also not affected by the surgery [36]. Male mice with surgical removal of the vomeronasal organ can still distinguish urine odors from other males and estrous females [37]. Thus, the idea that odorants are processed exclusively by the main olfactory system and pheromones by the accessory olfactory system is no longer universally accepted. Detailed electrophysiological studies in the main olfactory epithelium demonstrate that the main olfactory system is able to detect pheromones [38]. Taken together, our results support the proposal that the main olfactory system can detect and process pheromonal cues, but even more, that these cues can entrain rhythms of the olfactory system. Of course although previous evidence indicates that the MP is detected by the MOB, the effect observed in this structure may be due to other, of the many, components of mother’s milk.

However the importance of the main olfactory system in the detection of MP and the suckling of milk, this does not exclude a possible role of the accessory olfactory system. Perhaps an unidentified component in mother’s milk, different of the MP, is responsible of the above mentioned effect and it will be important to explore in detail, both their identity and their effect in the accessory olfactory system for the following reasons: 1) MP does not induce FOS in AOB [24], 2) suckling of milk induces a strong FOS-ir in mitral cell layer (present results), 3) Mitral cells are the output of the olfactory system to other regions of the brain [39].

In contrast to the apparent importance of the OB in the synchronization of rabbit pups, previous studies indicates that FAA persists after destruction of the nasal epithelium by intranasal zinc sulfate [5] and also after olfactory bulb ablation [6]. However, we consider that this does not rule out the role of olfactory cues because destruction of several other brain regions such as the arcuate nucleus [40], paraventricular nucleus and lateral hypothalamus [41], ventromedial nucleus [42], dorsomedial hypothalamic nucleus [43], paraventricular thalamic nucleus [44], among several others [2] also does not eliminate FAA. On the contrary, we consider that present results emphasize the biological relevance of the OB in food entrainment. **Future studies should explore whether this synchronization of OB plays a relevant role in FAA in the rabbit pup. In this regard, also will be important to explore if structures up- and downstream of the OB are also entrained to the restricted feeding schedule.**

On basis of the present and previous work, we conclude that the OB **can be entrained by food and perhaps plays an important role in conveying chemosensory entraining signals to induce FAA.** Finally, the present contribution confirms that the rabbit pup is an excellent model to explore the consequences of scheduled meals, as these subjects naturally ingest food just once a day with a circadian periodicity, thus providing a way to study FAA without drastic manipulations of food availability.

## Supporting Information

Figure S1 **FOS and PER1 expression in the Periglomerular and Granular cell layers of the Accessory olfactory bulb.** FOS-ir in periglomerular cell layer in AOB (Fig. S1, A, B) *Subjects nursed at 02∶00 h* Two-way ANOVA indicated that in nursed and fasted pups FOS expression in the periglomerular layer in AOB not varied significantly with group condition (F_1,55_ = 1.86, *P*<0.179) or time factor (F_6,55_ = 2.67, *P*<0.028), however, there was an interaction between feeding condition and time (F_6,55_ = 5.98, *P*<0.001; Fig. X). In the RF02∶00 group, the highest FOS expression at ZT16 was significantly different to values at ZT04, ZT08 (*P*<0.001 in both cases), ZT0, and ZT20 (*P* = 0.047 in both cases). Additionally, value at ZT1.5 was different from ZT04 (*P* = 0.005) and ZT08 (*P* = 0.013). In the RF-F02∶00 group, the highest expression of FOS at ZT04 was significantly different from ZT20 (*P* = 0.047). *Subjects nursed at 10∶00 h* Quantitative analysis indicated that in nursed and fasted pups FOS expression in the periglomerular layer in AOB varied significantly with group condition (F_1,55_ = 11.05, *P* = 0.002), time factor (F_6,55_ = 6.26, *P*<0.001), and the interaction between feeding condition and time (F_6,55_ = 10.28, *P*<0.001). In the RF10∶00 group, the highest value at ZT1.5 was significantly different than values at ZT0, ZT08, ZT12, ZT16, and ZT20 (*P*<0.001 in all cases). Additionally, the FOS expression at ZT04 was significantly different to values at ZT0 (*P* = 0.04), ZT12 (*P* = 0.005), ZT16 (*P* = 0.03), and ZT20 (*P* = 0.002). In RF-F10∶00, the highest expression of FOS at ZT16 was significantly different to values at ZT0, ZT1.5 (*P* = 0.01 in both cases), ZT04 (*P* = 0.03), and ZT12 (*P* = 0.023). PER1-ir in periglomerular cell layer in AOB (Fig. S1, C, D) *Subjects nursed at 02∶00 h* Two-way ANOVA indicated that in nursed and fasted pups PER1 expression in the periglomerular layer in AOB varied significantly with group condition (F_1,55_ = 28.43, *P*<0.001), time factor (F_6,55_ = 12.93, *P*<0.001) and the interaction between feeding condition and time (F_6,55_ = 21.43, *P*<0.001). In the RF02∶00 group, the highest PER1 expression at ZT20 was significantly different to values at ZT04 (*P*<0.001) and ZT08 (*P* = 0.047). In the RF-F02∶00 group, the highest expression of PER1 at ZT04 was significantly different to remaining time point values (*P*<0.001 in all cases). Additionally, value at ZT12 was different to value at ZT16 (*P* = 0.009). Also, ZT20 was significantly different to ZT16 (*P* = 0.015). *Subjects nursed at 10∶00 h* Quantitative analysis indicated that in nursed and fasted pups PER1 expression in the periglomerular layer in AOB varied significantly with group condition (F_1,55_ = 43.32, *P*<0.001), time factor (F_6,55_ = 7.44, *P*<0.001) and the interaction between feeding condition and time (F_6,55_ = 4.17, *P* = 0.002). In the RF10∶00 group, the highest value at ZT0 was significantly different to value at ZT1.5 (*P* = 0.05). In RF-F10∶00, the lowest expression of PER1at ZT1.5 was significantly different to values at ZT0, ZT08, ZT12, ZT20 (*P*<0.001 in all cases), ZT04 and ZT16 (*P* = 0.004). FOS-ir in granule cell layer in AOB (Fig. S1, E, F) *Subjects nursed at 02∶00 h* Two-way ANOVA indicated that in nursed and fasted pups FOS expression in the granule layer in AOB varied significantly with group condition (F_1,55_ = 37.7, *P* = 0.001), time factor (F_6,55_ = 20.45, *P*<0.001), and the interaction between feeding condition and time (F_6,55_ = 6.2, *P*<0.001). In the RF02∶00 group, the highest FOS expression at ZT1.5 was significantly different to values at ZT12, ZT16, and ZT20 (*P*<0.001 in all cases). Additionally, value at ZT0 and ZT08 were different from ZT20 (*P*<0.001in both cases), ZT12 and ZT16 (*P*<0.05 in all cases). In the RF-F02∶00 group, the highest expression of FOS at ZT04 and ZT08 was significantly different from ZT12 (*P*<0.01in both cases), ZT16 (*P*<0.001 in both cases), and ZT20 (*P* = 0.03 in both cases). Additionally, value at ZT1.5 was different to values at ZT12 (*P* = 0.032) and ZT16 (*P*<0.001). *Subjects nursed at 10∶00 h* Quantitative analysis indicated that in nursed and fasted pups FOS expression in the granule layer in AOB not varied significantly with group condition (F_1,55_ = 0.11, *P* = 0.745), but the time factor (F_6,55_ = 21.74, *P*<0.001) and the interaction between feeding condition and time (F_6,55_ = 38.61, *P*<0.001) varied significantly. In the RF10∶00 group, the highest value at ZT08 was significantly different to remaining time point values (*P*<0.001 in all cases). In RF-F10∶00, the highest expression of FOS at ZT0 and ZT16 were significantly different to values at ZT1.5, ZT04, ZT08, ZT12, ZT20 (*P*<0.001 in all cases). PER1-ir in granule cell layer in AOB (Fig. S1, G, H) *Subjects nursed at 02∶00 h* Two-way ANOVA indicated that in nursed and fasted pups PER1 expression in the granule layer in AOB varied significantly with group condition (F_1,55_ = 8.1, *P* = 0.007), time factor (F_6,55_ = 19.03, *P*<0.001), and the interaction between feeding condition and time (F_6,55_ = 4.63, *P* = 0.001). In the RF02∶00 group, the highest PER1 expression at ZT12 was significantly different to values at ZT0 (*P* = 0.003), ZT1.5, and ZT20 (*P = *0.04 in both cases). In the RF-F02∶00 group, the highest expression of PER1 at ZT12, was significantly different to remaining time point values (*P*<0.001 in all cases). *Subjects nursed at 10∶00 h* Quantitative analysis indicated that in nursed and fasted pups PER1 expression in the granule layer in AOB varied significantly with group condition (F_1,55_ = 4.96, *P* = 0.031), time factor (F_6,55_ = 10.05, *P*<0.001) and the interaction between feeding condition and time (F_6,55_ = 4.12, *P*<0.002). In the RF10∶00 group, the highest value at ZT08 was significantly different to values at ZT0, ZT20 (*P*<0.001in both cases), ZT1.5 (*P* = 0.002), and ZT16 (*P* = 0.021). In RF-F10∶00, the highest expression of PER1 at ZT12 was significantly different to values at ZT0, ZT1.5, ZT08, ZT16, and ZT20 (*P*<0.001 in all cases).
(TIFF)
