# Supplementary figures and images for: A Circadian Clock in the Olfactory Bulb Anticipates Feeding during Food Anticipatory Activity
Source: PLoS One. 2012 Oct 19;7(10):e47779. doi: 10.1371/journal.pone.0047779 (PMC3477144; doi:10.1371/journal.pone.0047779)

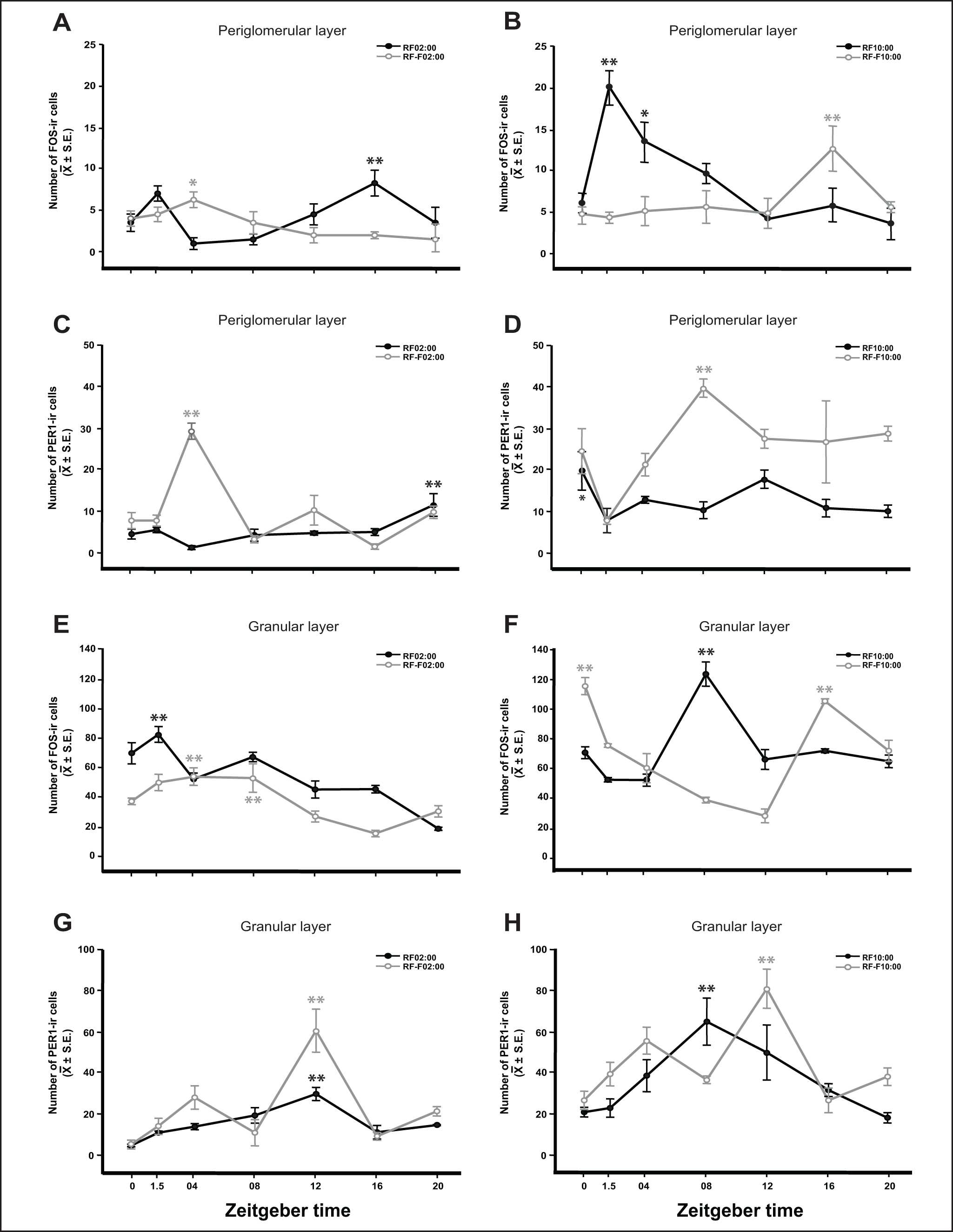

Supplement: Figure S1 — FOS and PER1 expression in the Periglomerular and Granular cell layers of the Accessory olfactory bulb. FOS-ir in periglomerular cell layer in AOB (Fig. S1, A, B) Subjects nursed at 02∶00 h Two-way ANOVA indicated that in nursed and fasted pups FOS expression in the periglomerular layer in AOB not varied significantly with group condition (F1,55 = 1.86, P<0.179) or time factor (F6,55 = 2.67, P<0.028), however, there was an interaction between feeding condition and time (F6,55 = 5.98, P<0.001; Fig. X). In the RF02∶00 group, the highest FOS expression at ZT16 was significantly different to values at ZT04, ZT08 (P<0.001 in both cases), ZT0, and ZT20 (P = 0.047 in both cases). Additionally, value at ZT1.5 was different from ZT04 (P = 0.005) and ZT08 (P = 0.013). In the RF-F02∶00 group, the highest expression of FOS at ZT04 was significantly different from ZT20 (P = 0.047). Subjects nursed at 10∶00 h Quantitative analysis indicated that in nursed and fasted pups FOS expression in the periglomerular layer in AOB varied significantly with group condition (F1,55 = 11.05, P = 0.002), time factor (F6,55 = 6.26, P<0.001), and the interaction between feeding condition and time (F6,55 = 10.28, P<0.001). In the RF10∶00 group, the highest value at ZT1.5 was significantly different than values at ZT0, ZT08, ZT12, ZT16, and ZT20 (P<0.001 in all cases). Additionally, the FOS expression at ZT04 was significantly different to values at ZT0 (P = 0.04), ZT12 (P = 0.005), ZT16 (P = 0.03), and ZT20 (P = 0.002). In RF-F10∶00, the highest expression of FOS at ZT16 was significantly different to values at ZT0, ZT1.5 (P = 0.01 in both cases), ZT04 (P = 0.03), and ZT12 (P = 0.023). PER1-ir in periglomerular cell layer in AOB (Fig. S1, C, D) Subjects nursed at 02∶00 h Two-way ANOVA indicated that in nursed and fasted pups PER1 expression in the periglomerular layer in AOB varied significantly with group condition (F1,55 = 28.43, P<0.001), time factor (F6,55 = 12.93, P<0.001) and the interaction [file pone.0047779.s001.tif]
